# Detection of Malawi polyomavirus sequences in secondary lymphoid tissues from Italian healthy children: a transient site of infection

**DOI:** 10.1186/s12985-016-0553-z

**Published:** 2016-06-10

**Authors:** N. Papa, N. Zanotta, A. Knowles, E. Orzan, M. Comar

**Affiliations:** Institute for Maternal and Child Health - IRCCS “Burlo Garofolo”, Via dell’Istria 65, 34137 Trieste, Italy; Medical Sciences Department, University of Trieste, Piazzale Europa 1, 34128 Trieste, Italy

**Keywords:** Malawi infection, Children, Lymphoid tissues, Route of transmission, Sites of persistence

## Abstract

**Background:**

The novel Malawi polyomavirus (MWPyV) was initially detected in stool specimens from healthy children and children with gastrointestinal symptoms, mostly diarrhea, indicating that MWPyV might play a role in human gastroenteric diseases. Recently, MWPyV sequences were additionally identified in respiratory secretions from both healthy and acutely ill children suggesting that MWPyV may have a tropism for different human tissues. This study was designed to investigate the possible sites of latency/persistence for MWPyV in a cohort of healthy Italian children.

**Methods:**

Specimens (n° 500) of tonsils, adenoids, blood, urines and feces, from 200 healthy and immunocompetent children (age range: 1–15 years) were tested for the amplification of the MWPyV LT antigen sequence by quantitative real-time PCR. Samples (n° 80) of blood and urines from 40 age-matched children with autoimmune diseases, were screened for comparison. Polyomaviruses JC/BK and Epstein-Barr Virus (EBV) were also tested as markers of infection in all samples using the same molecular technique.

**Results:**

In our series of healthy children, MWPyV was detected only in the lymphoid tissues showing a prevalence of 6 % in tonsils and 1 % in adenoids, although with a low viral load. No JCPyV or BKPyV co-infection was found in MWPyV positive samples, while EBV showed a similar percentage of both in tonsils and adenoids (38 and 37 %).

Conversely, no MWPyV DNA was detected in stool from babies with gastroenteric syndrome. With regards to autoimmune children, neither MWPyV nor BKPyV were detected in blood, while JCPyV viremia was observed in 15 % (6/40) of children treated with Infliximab. Urinary BKPyV shedding was observed in 12.5 % (5/40) while JCPyV in 100 % of the samples.

**Conclusions:**

The detection of MWPyV sequences in tonsils and adenoids of healthy children suggests that secondary lymphoid tissues can harbour MWPyV probably as transient sites of persistence rather than actual sites of latency.

## Background

The family of human polyomaviruses (PyVs), a group of small DNA viruses, has recently grown to include 13 members, raising the question of their potential role in humans, with and without manifest diseases [1–4].

PyVs seem to be harmless in immunocompetent individuals, but they can cause diseases in immunocompromised patients. The etiological role in human diseases has been established for 4 of the 13 human polyomaviruses, including tumors without productive viral replication and non-neoplastic productive viral infections.

The known human polyomaviruses BKPyV and JCPyV, display potential tumorigenic characteristics, although their role in human cancers remains controversial [5–7]. The only human PyV to have been recognized as potential oncovirus is the recently discovered Merkel cell polyomavirus (MCPyV), which seems to be a causal factor in Merkel cell carcinoma, an aggressive skin malignancy [8–11]. On the other hand, BKPyV, but also JCPyV, is associated with polyomavirus nephropathy (PVAN) in renal transplant recipients, and BKPyV is also associated with hemorrhagic cystitis in bone marrow transplant patients, kidney allograft dysfunction and potential graft loss [12–15]. JCPyV is the etiological agent of progressive multifocal leukoencephalopathy (PML) [16, 17], while the Trichodysplasia Spinulosa-associated polyomavirus (TSPyV) is thought to be the etiological agent of trichodysplasia spinulosa, also known as pylomatrix dysplasia, a rare skin disease characterized by the development of follicular papules and keratin spines [18–20].

Nothing is known on the possible role in human diseases of the newly discovered polyomaviruses WUPyV and KIPyV, the Saint Louis polyomavirus (STLPyV), the Malawi polyomavirus (MWPyV), the Human polyomavirus 12 (HPyV12) and the New Jersey polyomavirus (NJPyV),which have been detected in different human biological samples including naso-pharyngeal secretions [21–24], stool [25, 26], liver and vascular endothelial cells [4, 27]. However, HPyVs have been proposed as candidate causative factors in a number of diseases with unknown etiology. For instance, the association between these novel polyomaviruses and the primary mucosal melanomas has recently been investigated, albeit without significant results [28].

PyVs are ubiquitous viruses in the human population, with no less than 20 %, and up to 90 %, of the adult population carrying antibodies against these viruses, depending on the PyV strains, the geographic area and on the demographic characteristics of the study cohorts [29–31].

For almost all the members of the PyVs family, the route of intra-human transmission is still unknown. However, strong evidence indicates that JCPyV and BKPyV can establish latency in the genitourinary tract [32, 33], since episodes of self-limiting asymptomatic reactivation by intermittent urinary shedding or viremia can occur during severely impaired immune surveillance [34]. Nevertheless, other organ niches such as the gastroenteric trait [35], the lymphoid tissues [36, 37] and the central nervous system [38–41] have also been suggested as potential sites of latency.

The secondary lymphoid tissue has been proposed as a possible site of persistence for WUPyV, KIPyV and TSPyV by some authors, [42–44]. Moreover, MWPyV and STLPyV sequences were recently detected in 2 % of tonsillar swabs from children with chronic tonsillar disease, raising the question of how the lymphoid system may play a role in human polyomavirus infection and persistence [45].

Among new Polyomaviruses, particular attention has been dedicated, in Italy, to the Malawi polyomavirus since a sero-epidemiological survey on a large sample of subjects (from 1 to ≥ 80 years old), has documented the circulation of this virus with a trend of frequency similar to that reported for the human polyomaviruses JC and BK. Specifically, MWPyV seroconversion was shown to occur early in childhood, with 26.9 % of 1- to 2-year-old children testing seropositive, although seropositivity peaked (68.2 %) in 3- to 4-year-old children [46].

MWPyV was initially identified by shotgun pyrosequencing of DNA extracted from virus-like particles isolated from a stool sample collected from a healthy child in Malawi. MWPyV is an unenveloped icosahedral virion containing a small, closed circular double-strand DNA genome of 4927 bp. The complete DNA sequencing showed a MWPyV genome structure typical of the Polyomaviridae family, divided into three regions: the regulatory region, the early region and the late region. The early region encodes the large T antigen (LTAg) and small T antigen (STAg), while the late region is expressed after viral replication and encodes the structural proteins of the capsid VP1, VP2, VP3. The regulatory region contains the origin of replication and the promoter genes for transcription of the early and late regions [25].

Following, MWPyP was found in 2.3 % of fecal samples from patients with gastrointestinal diseases [25]. Subsequently, a novel highly divergent polyomavirus, provisionally named MXPyV, was identified by deep sequencing from diarrheal stool samples in Mexico. The whole-genome sequence of MXPyV was found to be nearly identical to that of polyomaviruses MWPyV and HPyV10, indicating that these three viruses are different variants of the same species [47]. The detection of MWPyV in stool of children with diarrhea, often without known etiology, raised the hypothesis that MWPyV might play a role in human gastroenteric diseases. It is also possible, however, that MWPyV has a tropism for other human tissues and is shed in stool as a mode of intra-human transmission [48]. Conversely, Rockett et al. reported the presence of MWPyV in respiratory secretions from acutely ill patients and in babies (<18 months old) with mild upper respiratory symptoms [49]. Therefore, the question of which virus excretion route drives human infection has not, as yet, been fully resolved.

One of the key points that need to be explored is whether MWPyV is a pathogen of humans, and if so what diseases are associated with its infection, or rather an environmental contaminant that transiently infects humans.

The present study was designed to investigate the site of latency/persistence of MWPyV in healthy children. Tonsil, adenoid, blood, urine and stool specimens from age-matched Italian healthy and immunocompetent children and from children with autoimmune disorders, were screened for this purpose.

JCPyV and BKPyV, which are known to be present with a high number of excreted virions in the urine and feces of immunocompromised subjects [33, 35] and EBV considered a lymphotropic virus, were also tested as markers of infection.

## Methods

### Subjects and samples

The demographic and clinical characteristics of 240 Italian Caucasian children (200 immunocompetent (healthy) children and 40 autoimmune children) of both sexes, aged 1 to 15 years and referred to the Pediatric Department of the Children’s Hospital “Burlo Garofolo” in Trieste, Italy, are detailed in Table 1.

**Table 1:** Main demographic and clinical characteristics of children cohorts

Healthy subjects	No. 200
Group 1	No. 100
Age in years, mean	5.4
Age categories, number (percentage)	
1–5 y	59 (59 %)
6–15 y	41 (41 %)
Female, number (percentage)	43 (43 %)
Diagnosis, number (percentage)	
Tonsils/ Adenoids hypertrophy	100 (100 %)
Group 2^a^	No. 100
Age in years, mean	1.9
Age categories, number (percentage)	
0–4 y	100 (100 %)
Female, number (percentage)	70 (70 %)
Diagnosis, number (percentage)	
Enterovirus	10 (10 %)
Norovirus	20 (20 %)
Autoimmune children	No. 40
Age in years, mean	8.5
Age categories, number (percentage)	
1–5 y	11 (27.5 %)
6–15 y	29 (72.5 %)
Female, number (percentage)	23 (57.5 %)
Diagnosis, number (percentage)	
Crohn Disease	13 (32.5 %)
Juvenile Rheumatoid Arthritis (JRA)	20 (50.0 %)
Ulcerative Rectocolitis	7 (17.5 %)

^a^Gastrointestinal disorder children

Among the healthy children, group 1 consisted of 59 children aged 1 to 5 years old, and 41 children aged 6 to 15 years old (mean age of group 1: 5.4 years). These subjects had undergone adeno-tonsillar surgery for hypertrophy of adenoids, defined as adenoids occupying over 80 % of the choanal space, and tonsils graded three (30/100) or four (70/100), according to Brodsky’s classification [50]. Four-hundred matched biological samples, including blood, urine, tonsil and adenoid tissues, were collected from these children.

Group 2, consisted of 100 healthy babies (mean age: 1.9 years; age range: 0–4 years old) affected by acute gastrointestinal pain and diarrhea. By the routine microbiological analysis, ten percent of these children tested positive for Enterovirus (10/100), 20 % for Norovirus (20/100), but all samples resulted negative for other gastrointestinal viruses including Rotavirus, Adenovirus, and Astrovirus.

The 40 autoimmune children (mean age: 8.5 years) included 11 aged 1 to 5 years old, and 29 aged 6–15 years old. These children were affected by inflammatory diseases including Crohn’s disease (13/40), Ulcerative Rectocolitis (7/40) and Juvenile Rheumatoid Arthritis (20/40). Twenty of them had been treated with biological drug monotherapy (Infliximab) because they were refractory to standard pharmacological treatment, but were clinically well at the time of sample collection (blood and urine).

### Sample processing and DNA extraction

Upon arrival in the laboratory, all specimens were processed for DNA automated extraction. Twenty mg of fresh tissue from tonsils and adenoids were diced into small pieces, suspended for digestion in a mixture composed by 400 μL of easyMAG lysis buffer (bioMérieux, France) and 40 μL of proteinase K (200 μg/mL), and incubated at 56 °C overnight. Stool specimens were dissolved in sterile water, vortexed and clarified by centrifugation at 6000 g for 10 min (min), and the supernatant recovered for extraction. Total available urines were collected in a 50 mL tube, centrifuged at 4500 g for 20 min and the pellet suspended in sterile water. Whole EDTA blood (100 μL) was diluted in easyMAG lysis buffer (900 μL) at room temperature for 10 min before extraction.

DNA isolation was performed using the NucliSenseasyMAG platform (bioMérieux, France), according to the manufacturer’s protocol. DNA was eluted to a final volume of 50 μL and stored at −80 °C until analysis.

### Virological molecular detection

Quantitative real-time PCR (qPCR) assay was performed, using TaqMan chemistry, to detect Malawi polyomavirus, employing the AB PRISM 7500 Sequence Detection System (Applied Biosystem, Milan, Italy). The specific primer pairs were designed to detect the viral large T protein as previously described [25]. Serial 10-fold dilutions ranging from 10^9^ to10^0^ copies of a positive control plasmid (plasmid K-p31, Addgene, USA) were performed to generate standard curves. Briefly, the total 25 μL PCR mixture was composed by 5 μL of extracted nucleic acid, 1 X universal TaqMan real-time PCR master mix (Applied Biosystem), 12.5 pmol of each primer and 4 pmol of the probe. The cycling conditions were: 50 °C for 2 min, 95 °C for 10 min and 45 cycles of 95 °C for 15 s followed by 60 °C for 1 min. A negative control (containing water instead of DNA templates) and one dilution of the plasmid as positive control were added to each run. Based on the standard curve, the MWPyV assay demonstrated a reliable detection limit of approximately five copies per reaction, yielded a linear regression R^2^ value of 0.99, and was 93 % efficient.

Multiplex TaqMan qPCR was also performed on the same samples to measure the viral loads of JCPyV and BKPyV and to simultaneously amplify the reference human cellular β-globin gene in order to determine the cell equivalents present in each sample and then to calculate the proportion of infected cells (Euro-RT Polyoma Panel kit; Eurospital Spa, Trieste, Italy). In addition, in lymphoid organs, human Epstein-Barr Virus (EBV), used as reference lymphotropic virus, was assayed by specific qPCR with TaqMan chemistry, using commercially available kits (Nanogen, Turin, Italy) according to manufacturer’s recommendations [51].

Finally, enteroviral-pathogens detection was performed using a commercially available kit for Enterovirus (Elite MGB Kit, Elitech Group, Puteaux, France), and by a qualitative EIA test (enzyme immunoassay) (Ridascreen, R-Biopharm, Darmstadt, Germany) for Norovirus, Adenovirus, Astrovirus and Rotavirus, following the supplier’s recommendations.

### Statistical analysis

The statistical analysis was performed using IBM SPSS Statistics 20. Fisher Exact Test was used to compare the frequencies of discrete variables. A *P* value ≤ 0.05 was considered as the threshold of statistical significance.

## Results

### Human polyomaviruses detection

In this series of children, the overall prevalence of MWPyV was 2.9 % (7/240). The infection was detected only in the healthy/immunocompetent cohort (mean age 6.8 years) where it reached a prevalence of 3.5 % (7/200). No difference according to gender was observed (*p* > 0.05) in these series.

With regards to the distribution of MWPyV infection, considering all types of biological samples, as shown in Table 2 the infection could only be detected in the lymphoid tissues of healthy children, and more precisely in tonsils (6 %) and in adenoids (1 %). Infections were characterized by low replication rates, typically ranging from 5 to 10 viral copies/reaction. The proportion of infected cells, expressed as the number of viral copies in the cells (% of infected cells: from 0.05 to 1.0) and calculated on the basis of the copies of β-globin (range: 3 × 10^3^ to 1 × 10^4^copies/reaction) assuming that one copy of the viral genome infects one cell, was constantly low. No JCPyV or BKPyV co-infection was found in MWPyV positive samples, while the EBV lymphotropic reference showed a similar percentage of infection both in tonsils and adenoids (38 and 37 % respectively).

**Table 2:** Prevalence of Malawi and JC/BK polyomavirus and EBV infections in biological samples from healthy and autoimmune children

	Samples			Polyomavirus No. (%)		Ref. virus No. (%)
		Total No.	*MWPyV*	*JCPyV*	*BKPyV*	*EBV*
Healthy children						
No. 200	Tonsil	100	6 (6 %)	0	0	38 (38 %)
	Adenoid	100	1 (1 %)	0	0	37 (37 %)
	Blood	100	0	0	0	12 (12 %)
	Urine	100	0	0	0	–
	Stool^a^	100	0	0	0	–
	Total No.	500	7 (1.4 %)	0	0	–
Autoimmune children						
No. 40	Blood	40	0	6 (15 %)	0	2 (5 %)
	Urine	40	0	40 (100 %)	5 (12.5 %)	–
	Total No.	80	0	46 (57.5 %)	5 (6.25 %)	–

^a^30 with acute gastrointestinal symptoms with a diagnosis of Enterovirus 10/30 and Norovirus 20/30 infections

No MWPyV DNA was detected in stool specimens from babies with gastroenteric syndrome with or without diarrhea.

With regards to autoimmune children, neither MWPyV nor BKPyV genomic sequences were detected in blood, while JCPyV viremia was observed in 15 % (6/40) of the samples (geometric mean viral load of 3.9 × 10^3^ copies/mL) recovered from children in therapy with Infliximab. The lymphotropic EBV was also present in 5 % (2/40) of the blood samples. Urinary BKPyV shedding was observed in 12.5 % (5/40) (geometric mean viral load of 3.7 × 10^3^ copies/mL) of the children, while JCPyV shedding was detected in 100 % of the urine samples (geometric mean viral load of 2.5 × 10^6^copies/mL).

## Discussion and conclusions

Several studies have reported the widespread distribution of MWPyV in different areas of the world, including Africa, USA, Asia, Australia and South America [25, 45, 48, 49, 52]**.** A recent MWPyV seroprevalence survey on the Italian general population has documented, for the first time, the circulation of this polyomavirus also in this geographic region [46].

The detection of MWPyV sequences in stool and respiratory samples from healthy children in different geographical areas, suggests that the establishment of the primary infection is likely to have taken place at an early age, and that the gastrointestinal and/or respiratory tracts may be sites of viral persistence. However, the relationship of MWPyV with immunity is poorly understood, and this can be explained by the absence of a recognizable clinical condition and of a definitive identification of the human tissues where it latently persists.

In the present study, based on the natural history of other human polyomaviruses, tonsil and adenoid biopsies and blood and urine specimens were screened in healthy and autoimmune children, to investigate possible sites of MWPyV persistence.

The results from this series, show an overall frequency of MWPyV infection of 2.9 % which increases slightly to 3.5 %, when only the cohort of healthy children is considered. The discrepancy between the virus detection rate and the high seroprevalence rate (from 26.9 % to around 52 %) found in Italian healthy children of similar age [46], suggests that MWPyV may infect a host niche that has not yet been properly identified.

The association of MWPyV with gastroenteric symptoms (e.g., diarrhea) has suggested a possible role for this virus in intestinal disorders, either acting alone or in synergy with enteric pathogens [25, 53]. For this reason, we tested stool specimens from healthy babies with diarrhea, 30 % of whom had a diagnosis of Entero- and Norovirus infections, but yielded no positive results. This contrast in data may be due to the small number of samples we analyzed (n°100) compared to the published St. Louis series (n°514), and to the low prevalence rate of infection (2.3 %) registered in that study [25]. In addition, the frequency and duration of MWPyV stool shedding after infection/reactivation has not, as yet, been established with certainty, and this may have led to an underestimation of infection in St. Louis study.

The results from this study seem to be in line with those from a recent investigation by Rockett et al. in which MWPyV was associated with upper respiratory infection symptoms in >33 % of sole detection episodes, and only rarely with gastrointestinal symptoms [49]. Moreover, in a previous Australian study by the same author, MWPyV viremia and viruria were not observed in either the healthy or the autoimmune group, indicating that there was no systemic involvement of peripheral lymphocytes [53]. Malawi DNA is more likely to be detectable in blood during the primary infection rather than during reactivation, the latter being an event that depends on the specific biological features of the virus, on local stimuli in infected tissues and on the level of immunosuppression of the host [54].

The present study shows that MWPyV can infect lymphoid cells from healthy children, although at a lower rate (7 %) than lymphotropic EBV (37.5 %) and WUPyV (34.9 %) [43], thereby supporting the hypothesis of the transient nature of these tissues as sites of infection. These data appear to be in contrast with those by Peng et al. [45], which highlighted the role of tonsils as sites of persistence also for MWPyV, despite the low rate of detection (2 %) and the likelihood oral-pharyngeal contamination during swab sampling of the mucosal surfaces of palatine tonsils.

Although previous studies have shown that different polyomavirus strains can coexist in the same organ of the same host, there is no evidence of BKPyV, JCPyV or EBV co-infection in these tissues [55–57].

The association between diminished immunity and reactivation is less clear for this novel human polyomavirus. In our study, MWPyV was undetectable in the urine and in the blood of autoimmune children, while, in agreement with other published data, urinary shedding of BKPyV and JCPyV was confirmed. Moreover, JCPyV reactivation or subclinical infection was detected in the blood of 6 children treated with biological drug, confirming the risk of developing PML as a result of long-term of monoclonal therapy [58–60].

In conclusion, in this series of healthy immunocompetent Italian children, no trace of MWPyV infection was found in biological samples indicated by previous studies as possible vehicles of intra-human transmission. Conversely, our data demonstrated that the secondary lymphoid tissues can actually harbour MWPyV. However, only very few cells proved to be infected suggesting that these tissues are unlikely to be the preferred site of latency, at least in this series of children.

Nevertheless, the high MWPyV seroprevalence found in young children from this geographic area, and the in vitro ability of MWPyV to bind to cellular tumor suppressor factors and to promote chronic infection [52], seem to support the hypothesis that infection/reactivation and transmission of MWPyV may have clinical consequences only in severely immunocompromised hosts. This observation warrants further investigations in organ transplant recipients and AIDS and cancer patients.

## Abbreviations

BKPyV, BK polyomavirus; EBV, Epstein Barr Virus; EIA: enzyme immunoassay; HPyV12, human polyomavirus 12; JCPyV, JC polyomavirus; KIPyV, KI polyomavirus; MCPyV, Merkel cell polyomavirus; MWPyV, Malawi polyomavirus; NJPyV, New Jersey polyomavirus; PML, progressive multifocal leukoencephalopathy; PVAN, polyomavirus associated nephropathy; PyV, polyomavirus; real-time qPCR, quantitative real-time PCR; STLPyV, Saint Louis polyomavirus; TSPyV, TrichodysplasiaSpinulosa-associated polyomavirus; WUPyV, WU polyomavirus
